# Carbon Nanotube-Based Chemiresistive Sensors

**DOI:** 10.3390/s17040882

**Published:** 2017-04-18

**Authors:** Ruixian Tang, Yongji Shi, Zhongyu Hou, Liangming Wei

**Affiliations:** Key Laboratory for Thin Film and Microfabrication of the Ministry of Education, School of Electronic Information and Electrical Engineering, Shanghai Jiao Tong University, 800 Dongchuan Road, Shanghai 200240, China; sugarapple@sjtu.edu.cn (R.T.); syj.sjtu.edu.cn@sjtu.edu.cn (Y.S.); zhyhou@sjtu.edu.cn (Z.H.)

**Keywords:** carbon nanotubes, chemiresistive, sensors, functionalization

## Abstract

The development of simple and low-cost chemical sensors is critically important for improving human life. Many types of chemical sensors have been developed. Among them, the chemiresistive sensors receive particular attention because of their simple structure, the ease of high precise measurement and the low cost. This review mainly focuses on carbon nanotube (CNT)-based chemiresistive sensors. We first describe the properties of CNTs and the structure of CNT chemiresistive sensors. Next, the sensing mechanism and the performance parameters of the sensors are discussed. Then, we detail the status of the CNT chemiresistive sensors for detection of different analytes. Lastly, we put forward the remaining challenges for CNT chemiresistive sensors and outlook the possible opportunity for CNT chemiresistive sensors in the future.

## 1. Introduction

We employ chemical sensors to detect target analytes which are of vital importance in our life. For example, industrial gas emissions and automobile exhaust which are closely related with air quality need to be detected. In the medical field, a variety of biomolecules must be monitored to help diagnose diseases. In the field of national defense and the military, some dangerous materials such as explosives and nerve gases must be detected. No matter what kind of transduction mechanism is used, a sensor must meet various requirements, including high sensitivity, excellent selectivity, stability, low power consumption, ease of use, and a long service life. 

**Figure 1:**
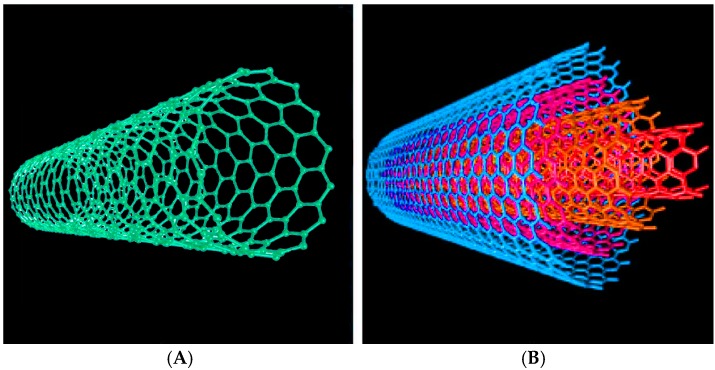
Schematics of an individual (**A**) SWCNT and (**B**) MWCNT. Cited from Reference [9] with permission.

The sensing materials used are the key components in chemical sensors. During the last decade, carbon nanotubes (CNTs) have be considered ideal sensing materials for chemical sensors because of their unique properties. CNTs, discovered by Iijima in 1991 [1], as shown in Figure 1, are categorized as single-wall carbon nanotubes (SWCNTs) and multi-wall carbon nanotubes (MWCNTs). SWCNTs consist of single rolled layers of graphene, with diameters ranging from 0.4 nm to 6 nm, and their length reaching a few microns. MWCNTs consist of multiple rolled layers of graphene, and their diameters range from several nanometers to tens of nanometers. Because of the strong π-π interaction between CNTs, CNTs tend to aggregate together to form tube bundles. 

CNTs show excellent mechanical and electronic properties. For example, the elastic modulus of CNTs is above 1 TPa, which is similar to diamond, and the strength is approximately 20 times that of carbon fiber, 100 times that of steel, but with a density that is one-sixth that of steel. Due to the remarkable conjugation effect, cylindrical carbon nanotubes have good electrical conductivity. Approximately one-third of CNTs are metallic, and the remaining CNTs are semiconducting according to their diameter and helix angle. Metallic CNTs can be used as single electron transistors, while semiconducting CNTs can be used for constructing field effect transistors (FETs).

**Figure 2:**
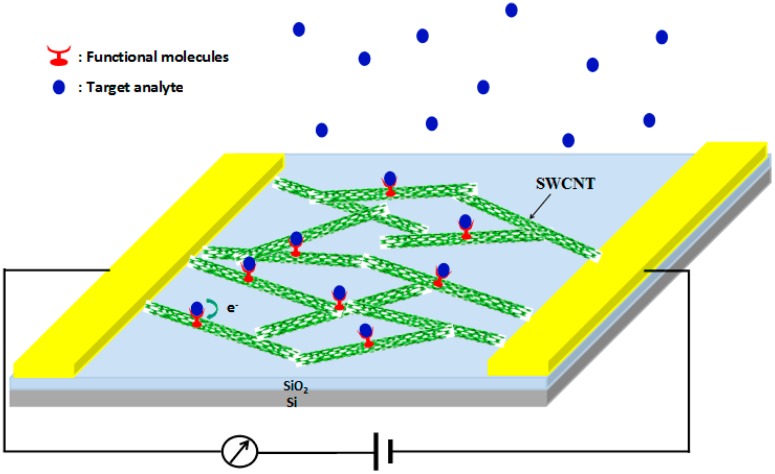
Schematic of configurations of CNT chemiresistive sensor.

CNTs are ideal sensing materials for compact, low power and portable sensing devices. SWNTs are almost entirely composed of surface atoms, and are expected to exhibit excellent sensitivity toward adsorbates. CNT sensors offer significant advantages over conventional metal oxide-based sensor materials in these aspects: room temperature operation, high sensitivity and easy miniaturization of construction for massive sensor arrays. SWCNTs possess high aspect ratio, good environmental stability, excellent mechanical and electronic properties, and ultrahigh surface to volume ratios. Since Kong et al. first made use of SWNT field-effect transistors (FETs) to detect NO_2_ and NH_3_, SWCNT-based sensors have been successfully used to detect a variety of gases or chemical vapors, such as NO_2_, O_2_, HCl, explosive gases and volatile organic compounds (VOCs). Multiple types of CNT-based sensing devices have been developed, such as chemiresistors, chemicapacitors and field-effect-transistors (FETs). Chemiresistors are very attractive because of their simple structure, low cost and the ease of high precision measurement. Figure 2 shows a schematic of a functionalized CNT-based chemiresistive sensor. In this chemiresistive sensor, the CNTs are used as conducting channels between the electrodes. The conductance changes between two electrodes are measured to investigate the sensing response. There have been some reviews concerning carbon nanotube-based sensors [2,3,4,5,6,7,8]. This review also mainly focuses on carbon nanotube-based chemiresistive sensors.

## 2. Functionalization of CNTs for Chemiresistive Sensors

CNTs present excellent properties when used as sensing materials. However, pristine CNTs always show weak responses and low selectivity toward specific gas molecules due to the weak interaction between CNTs and analyte molecules, so there is a need to functionalize CNTs to improve both the sensitivity and the selectivity of SWNT sensors [10,11,12,13,14,15,16,17,18,19,20,21,22,23]. Noncovalent functionalization and covalent functionalization are the two primary methods to functionalize the surface of CNTs [24,25,26,27]. The non-covalent functionalization is based on supramolecular complexation via wrapping and adsorptive forces (e.g., π-stacking interactions and Van der Waals forces). Covalent functionalization covalently links the small organic molecules, polymers or metal nanoparticles on the surface of CNTs. These functionalized CNT sensors always exhibit high sensitivity and high selectivity toward target analytes.

The covalent functionalization of CNTs can be performed by using the oxygen-containing groups (OH, COOH, et.al.) on the surface of CNTs to covalently link functional molecules on the surface of the CNTs. Some highly reactive species, such as free radicals, carbenes and diazonium ions, can directly react with the carbon atoms of CNTs to form covalent bonds. Non-covalent functionalization does not damage the structure of the sidewall and the perfect electronic properties can be retained. Organic polymers are commonly used as sensing films in gas sensors. The sensing mechanism of organic polymer-functionalized CNT sensors is that when they interact with analytes, the chemical or physical properties of the polymers change, which can disturb the conductance of CNTs. Conducting polymers (e.g., polypyrrole, polyaniline, polythiophene) [28] are used commonly because they show semiconducting or even metallic properties. The electron density of the conducting polymer might be changed when they absorb analytes. Metal nanoparticles have a broad range of properties in several fields such as electronics, chemistry and physics [29]. Previous works indicated that obvious charge reorganization occurs when CNTs interact strongly with metallic clusters [5,30,31,32,33]. The CNTs functionalized with metal nanoparticles show high sensitivity and selectivity toward some analytes, such as H_2_, CO, et al. Additionally, the sensors based on metal nanoparticles can work at a higher temperature or in a harsher environment compared to the organic molecule-functionalized CNT sensors. These metal nanoparticles include gold, silver, copper, cobalt, nickel and other metal elements [34].

## 3. Main Performance Parameter of CNT Sensors

**Figure 3:**
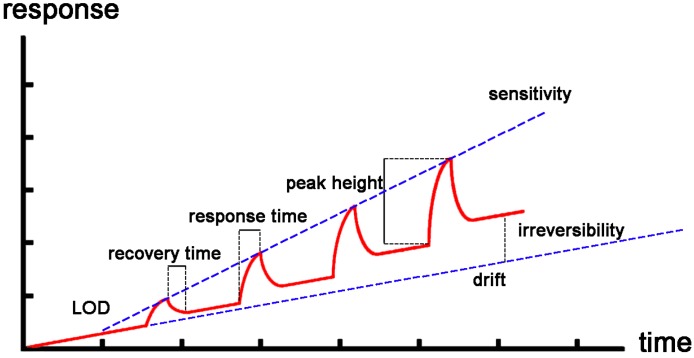
Selected performance parameters of a device which is exposed to a rising concentration of analytes. Cited from Reference [24] with permission.

A sensor is a device that can respond to an external stimulus and give a recognizable signal. The external stimulus will change the conductance, capacitance, mass or other properties of the sensing material which are easy to be measured. A sensor’s performance is characterized by a different kind of parameters (Figure 3) [24]. These main parameters include: sensitivity, selectivity, stability, drift, response time, reversibility and so on. The sensitivity is closely related with the detection limit, which is the minimum value of an analyte that can be measured by the sensor. The lower the detection limit, the higher the sensitivity. The selectivity shows the sensor’s capability to distinguish the target analytes among a number of interferents. Many sensors require functionalization with recognition elements to increase the selectivity. Stability means the sensor’s capability to give the same response signal for an identical stimulus over time. A good sensor should remain stable over the lifespan of the sensor. Drift occurs when the response of a sensor changes even if the surroundings are fixed. It can lead to false or uncertain response signals. Response time is the time the sensor needs to increase up to the 90% of its final stable output after introducing the analyte. Comparably, recovery time is the time the sensor takes to decline to the 10% of its baseline after the analytes are removed. Other parameters such as hysteresis, deadtime, risetime and dynamic range have also been used to characterize the sensor performance, which have been full discussed in [23] by Fennel et al.

## 4. Sensing Mechanism of Chemiresistive Sensors

The chemiresistive sensor is one of the most commonly used sensors in which CNTs can be employed as the conducting channel. Two methods are generally used to position CNTs between two electrodes. CNT networks can be deposited between the electrodes by solid transfer, printing, spraying, chemical vapor deposition (CVD) growth or drop-casting. During the analysis process, the conductance between two electrodes is measured to investigate the sensing response. Because CNTs are composed almost entirely of surface atoms, a very small change in the chemical environment around the CNTs will result in measurable conductance change. 

**Figure 4:**
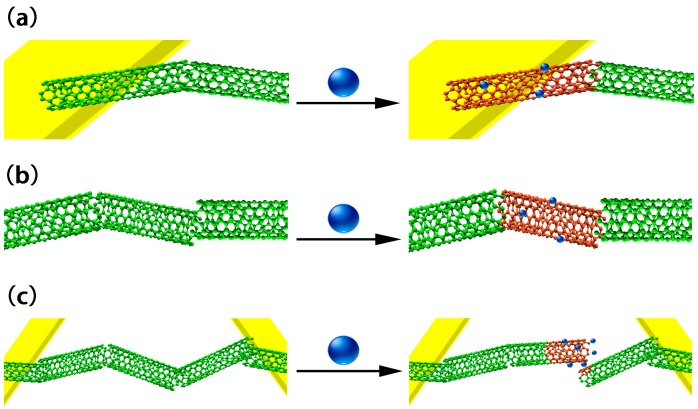
Three ways that analytes change the conductance of CNTs. (**a**) SWNT-electrode junction; (**b**) charge transfer among the SWNT and analytes; (**c**) intertube junction. Cited from [24].

The CNT sensing mechanism has been extensively discussed by Fennell et al. [24]. Briefly, the absorption of analytes onto the CNT surface can change the conductance via the following factors (Figure 4): modulation of the Schottky barrier at electrode-CNT junctions, charge transfer between and CNT and the analytes, and increases in the CNT–CNT junction distance. For example, CNTs are p-type semiconductor under ambient conditions. The electrons donated into the valence band of CNTs by absorption of analytes will result in a decrease of hole concentration, causing a decrease in conductance. Conversely, electrons withdrawn from p-type SWCNTs will lead to an increase of the hole concentration in the CNT, resulting in an increase in conductance. The conductance change can also result from reducing the CNT charge mobility by introducing scattering sites. If some analytes are absorbed on the CNT-metal interface, the conductance might change by modifying the Schottky barrier. Because individual CNTs are not long enough to form conducting channels, the conducting channels are generally formed via connection by many CNTs. If the analytes are absorbed on the intertube junction, the conductance of CNTs can also be changed by disturbing the intertube junctions.

## 5. Nitrogen Dioxide (NO_2_) Chemiresistive Sensors

It is important to detect NH_3_ and NO_2_ because they are harmful to human health and the environment [35]. Various CNT-based sensors have emerged to monitor these two toxic gases [36,37,38,39,40,41,42]. Valentini et al. [43] demonstrated a chemiresistive gas sensor for sensing NO_2_. They manufactured CNTs by ratio-frequency plasma-enhanced chemical vapor deposition (rf-PECVD) technology. The CNT films and a thin film of Ni catalyst were deposited on the Si_3_N_4_/Si substrate together. Platinum interdigitated electrodes and a back-deposited thin film platinum heater were placed on the back of the substrate to adjust the operational temperature. To improve the sensitivity (S), they creatively adopted three different thermal treatment protocols. As a result, when the gas concentration of NO_2_ was 100 ppm, the sensitivity (S) improved from 3.3% (after the first thermal treatment) to 56% (after the second treatment). These sensors are steadier than those metal oxide sensors which suffered from the grain and unreliable chemical properties of surface metal ions. Furthermore, the film resistance changed from 0.1 kΩ to 2 kΩ, which is remarkably lower than metal oxide sensors whose resistance is in the range of 500 kΩ to 50 MΩ. Thus, a lower driving voltage is obtained, which can improve the convenience and simplicity of electronic circuitry.

Sharma et al. [44] prepared CuPcOC_8_/MWCNTs-COOH hybrid material to detect several dangerous gases (e.g., NO_2,_ NH_3_ and Cl_2_) at 150 °C. MWCNTs-COOH was functionalized with CuPcOC_8_ (CuPcOC8 = 2, 3, 9, 10, 16, 17, 23, 24-octakis (octyloxy)-29H, 31H) to form CuPcOC_8_ /MWCNTs -COOH hybrid material. The π-π stacking interaction played an important role in this process to form charge transfer conjugate. Gaikwad et al. [45] reported a conducting polythiophene-SWNTs based chemiresisitive sensor for the monitor of NO_2_. In this sensing film, SWCNTs were aligned by using AC dielectrophoretic technique and the charge controlled potentiostatic deposition was used as a method to functionalize the surface of SWNTs. The sensor has a low cost and a good response for its LOD as low as 10 ppb. 

## 6. Ammonia (NH_3_) Chemiresistive Sensors

Wang et al. [46] developed a MWCNT gas sensor by using the microwave plasma-enhanced chemical vapor deposition method to detect gaseous NH_3_. In this sensor, the nanotubes were laid under the electrodes. This sensor is suitable for operating at room temperature. The sensitivity is approximately a linear function of gas concentration. The results showed that, the response time was ~180 s. However, due to the stronger binding of NH_3_ to CNTs, it took more than 6 hours to return to the original state in vacuum condition.

Mirica et al. [47] developed a gas sensor based on SWCNTs for sensing NH_3_. They fabricated the chemiresistive gas sensor by mechanical abrasion of compressed powders of sensing materials (SWCNT) on the fibers of cellulose. This sensor’s detection limit could be as low as 0.5 ppm. Cellulose paper was used as the substrate because it is inexpensive, compatible with certain forms of printing and surface-processing technologies and appropriate for abrasion-based writing and drawing with graphite and wax-based pencils. This SWCNT chemiresistive sensor showed the ability to be flexible, stackable, commercially available and potential wearable.

Rigoni et al. [48] presented a SWCNT chemiresistive gas sensor by a “drop and casting” method. In this method, the SWCNT bundle layers were carefully prepared and selected by dielectrophoresis-assisted deposition or by sonication of layers deposited by drop casting. The sensor worked at room temperature and possessed an enhanced sensitivity to NH_3_ with a detection limit of 3 ppb. The enhanced sensitivity was ascribed to the removal of loose agglomerates with poor electrical contact and a more efficient transport of carriers (transferred to the CNT bundles upon interaction with the reducing NH_3_ molecules), from the topmost SWCNTs to the underlying electrodes. It was shown that the dielectrophoresis method made SWNTs more aligned compared with disordered SWCNTs and provide higher sensitivity, which is consistent with other studies.

Some functionalized CNT sensors have also been developed. For example, He et al. [49] prepared polyaniline (PANI)-coated MWCNTs via in situ polymerization for the detection of ammonia. A fast response and a good reproducibility were showed at room temperature. A linear response was obtained when the concentration of ammonia ranging from 0.2 ppm to 15 ppm. Nguyen et al. [50] fabricated Co-functionalized MWCNT based sensors for detection of NH_3_. At room temperature, the sensor exhibited good repeatability and high selectivity towards NH_3_.

Lee et al. [51] reported a chemical sensor with an excellent performance for NH_3_ monitor. The sensor is transparent and flexible. It is prepared by functionalized SWCNTs with Au nanoparticles. An obvious resistance change of the Au nanoparticles-decorated SWCNT film was observed when it is exposed to NH_3_. A high sensitivity is obtained and the LOD is about 255 ppb. It is believed that the electron donation of absorbed NH_3_ on the Au nanoparticles adds electronic density and hole-electron recombination, leading to a reduction of the hole current through the SWCNTs.

Liu et al. [52] reported a chemiresistive sensor to detect amines (including NH_3_) by functionalized SWCNTs with the cobalt meso-arylporphyrin complex (Co (tpp)) ClO4. This sensor exhibits an excellent selectivity and selectivity toward amines, including NH_3_, among various analytes (e.g., CO, isoprene, hexane and so on). The magnitude of the chemiresistive response to ammonia could be improved through changes in the oxidation state of the metal, the electron-withdrawing character of the porphyrinato ligand, and the counter anion. It suggested that the charge transfer was the main component of the signal transduction of the system. They applied these chemiresistors to detect various biogenic amines (i.e., putrescine, cadaverine) and in the monitoring of spoilage in raw meat and fish samples (chicken, pork, salmon, cod) over several days.

**Figure 5:**
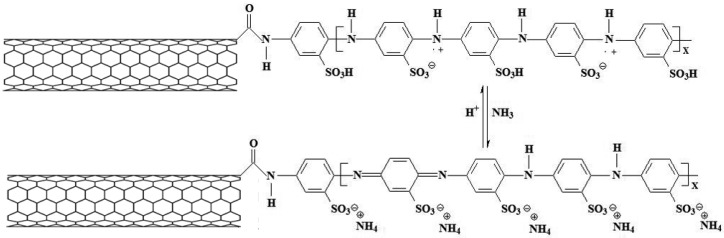
Interaction of SWNT-PABS with NH_3_. Adapted from Reference [53].

Bekyarova et al. [53] discovered a new method to functionalize SWCNTs which enhanced the performance of the sensor in NH_3_ detection by covalently attaching poly (*m*-aminobenzene sulfonic acid) on SWCNTs (SWNT-PABS) (Figure 5). It is believed that the sensing mechanism is mediated by the PABS chemically attached to SWCNTs. When NH_3_ interacts with PABS, the electronic structure of PABS is changed via the deprotonation of PABS which induces hole depletion and a reduced conductance of the SWCNT-PABS. The SWCNT-PABS devices presented more than two times higher resistance change compared with pristine SWCNTs. What’s more, the resistance will recover immediately if NH_3_ is taken off from nitrogen. The limit of detection is 5 ppm. 

Zhang et al. [54] demonstrated an electrochemical functionalization of SWCNT with polyaniline (PANI). On-line detection of NH_3_ is obtained by the PANI-SWNT network based sensors. Its sensitivity is up to 2.44% ΔR/R per ppm NH_3_, which is 60 times more than pristine SWNTs. The LOD is 50 ppb and the reproducibility is good upon repeated exposure. The recovery time is in hours and the response time is in minutes. Its sensitivity is high when the temperature is low. Lone et al. [55] demonstrated an efficient chemiresistive sensor with high sensitivity and fast response. A PECVD technique was used to grow vertically and uniform SWCNTs on an iron-deposited Si substrate at 550 °C. Proper gold contacts are made to fabricate the sensor. The response of the sensing device was measured by exposing the sensor to ammonia for five cycles at room temperature. The concentration of ammonia was 200 ppm. After removing the ammonia, the as-grown SWCNT-based sensing device recovered to 80% in a few minutes. Table 1 summarizes the sensing performance of selected NO_2_ and NH_3_ CNT-based chemiresistive sensors.

## 7. CNT-Based Hydrogen (H_2_) Chemiresistive Sensors

Hydrogen (H_2_) is a dangerous gas. When the concentration of hydrogen is just 4% in the air, explosions may occur. Pristine CNTs exhibit few advantages in detecting hydrogen because of the weak interaction between CNTs and H_2_, so the functionalization of CNTs is always necessary when CNTs are used to detect H_2_. Sayago et al. [56] evaluated a Pd-functionlized SWCNT gas sensor for the detection of hydrogen. It was found that the resistance increased sharply after the sensor was exposed to 0.1%–2% hydrogen. Pd-MWCNTs were also employed as sensing materials to fabricate a flexible and robust chemiresistor for hydrogen detection [57] that was used to detect 20 ppm hydrogen in a nitrogen environment. A layer of Pt was introduced to the Pd-MWCNT-based chemiresitor and the LOD was decreased to 5 ppm. In an air environment, the LOD of the Pd-MWCNT chemiresistor was 2000 ppm, compared to 400 ppm for a Pt-Pd-MWCNT. It is clearly that the Pt- Pd-MWCNT chemiresistor has much better sensitivity. 

PANI-coating MWCNTs are employed as sensing materials for hydrogen detection with high sensitivity [28]. The MWNT-PANI composite films presented a higher sensitivity toward hydrogen compared to bare PANI. Li et al. [58] fabricated a highly sensitive and selective sensor for hydrogen detection, with SWCNT/chitosan hybrid composite used as sensing film. The high sensitivity is ascribed to the chitosan which interacts with the hydrogen molecules selectively, but filters out the other gas molecules. The interaction between hydrogen molecules and -OH, -NH_2_ groups in chitosan provide a great contribution to the improvement of the sensor properties. A hybrid carbon nanostructrue include MWCNTs and graphene was used to detect hydrogen [59]. The sensor presented an increased sensitivity of 17% when the concentration of hydrogen in air is as low as 4 vol. %.

## 8. CNT-Based Greenhouse Gases Chemiresistive Sensors

Greenhouse gases (e.g., water vapor, carbon dioxide, methane, Freon) are the main causes of global warming. CO_2_ and methane are main compositions of greenhouse gases. CO_2_ is an exhaust gas of almost all industries and a metabolite of living beings. It becomes toxic when the level of CO_2_ is above 5%. Thus, the detection of CO_2_ is necessary both for the environment and human health. CH_4_ is a kind of colorless, odorless, explosive gas and its sources could be biogas digesters, coal mine gases, oil field gases and so on. In addition, CH_4_ is more powerful than CO_2_ in terms of its greenhouse effect. Thus, more attention should be paid to the detection of CH_4_. However, pure SWCNTs show no sensing ability toward methane, so the detection of methane focuses on functionalized CNT sensors [60].

Li et al. [61] fabricated a highly sensitive and selective chemiresistive CO_2_ sensor with a LOD as low as 500 ppt. The gas sensor was based on a uniform PIL/SWNTs film which is prepared using poly(ionic liquid) (PIL)-wrapped SWCNTs. Several advantages are shown for the sensor: high sensitivity and selectivity, reproducibility, and resistance to relative humidity. It is believed that when BF_4_ anions (belonging to PIL) on the surface of SWCNTs encounter CO_2_ molecules, charge transfer from BF_4_ to CO_2_ will reduce the whole electron-donating effect of (BF_4_) to carbon nanotubes, thus increasing the hole population in SWNTs, leading to a decrease in the resistance of the PIL wrapped SWNTs sensor.

Sainato et al. [62] demonstrated a MWCNTs/ZnO composite-based chemiresistive sensor to detect methane. ZnO was deposited as the functionalizing material to pretreat the surface of MWCNTs by atomic layer deposition (ALD). Crystalline quality improvement and functionalization strength are achieved via optimizing the ALD temperature. The LOD is 10 ppm. Three key factors were considered to account for the parts per million level CH_4_ detection: (1) strong relative resistance change of ZnO nanoparticles to low concentration level of CH_4_; (2) energetically favorable electron transport at MWCNTs/ZnO junction, and (3) strong electrical current modulation potential due to ballistic transport of electrons through the MWCNTs. Table 2 summarizes the sensing performance of selected H_2_/CO_2_/methane CNT-based chemiresistive sensors.

## 9. CNT-Based Volatile Organic Compound (VOC) Chemiresistive Sensors

It is reported that volatile organic compounds (VOCs) may damage the respiratory system and threaten human health. Therefore, various methods are employed to analyze VOCs [63,64]. Pophyrins are functional molecules that can be used to functionalize CNTs for sensors [65]. Sarkar et al. fabricated a SWCNTs−poly(tetraphenylporphyrin) hybrid through an electrochemical route and used this hybrid sensing material to detect acetone vapor [66]. The conductivity of pophyrins is poor, but it can be improved by fabricating a hybrid SWCNTs-poly(tetraphenylporphyrin) device. This sensor exhibited a wide dynamic range for acetone vapor sensing from 50 ppm to ~230,000 ppm with a LOD of 9 ppm. This sensor exhibited a good stability of over a period of 180 days. The field-effect transistor studies indicated electrostatic gating dominated the sensing mechanism. 

Shirsat et al. [67] presented a chemiresistive sensor array to detect VOCs. The sensor array combined SWCNTs with free-base and metaloporphyrins. The sensing performances depend on the types of central metal and the functional groups. The results show that free-base and metal (e.g., ruthenium and iron) substitution with octaethyl- and tetraphenylporphyrins provide good selectivity and sensitivity towards various VOCs tested. The sensing mechanism of the SWNT-Ru (octaethylporphyrin) device is governed by the electrostatic gating effect, and that of SWNT-Fe (tetraphenylporphyrin) is electrostatic gating combined with Schottky barrier modulation. This sensor provided a possibility for electronic nose implementation using a highly dense nanosensor array. Liu et al. [68] presented an array of chemiresistive sensors non-covalently functionalized with metalloporphyrin-SWCNT for VOCs detection. This functionalized SWCNT sensor has the ability to classify five different types of VOCs (alkanes, ketones, alcohols, aromatics, amines). The origins of the conductance changes in this sensor can be multiple, such as electrostatic gating effects, solvation of the metalloporphyrins, etc.

Formaldehyde is the most common and best-known indoor air pollutants. It is a Group 1 carcinogen for human beings and also irritates the mucosa of the upper respiratory tract by inhalation, so the detection of formaldehyde is of vital importance. We developed a chemiresistive sensor based on tetrafluorohydroquinone (TFQ)-functionalized semiconducting SWCNT networks for highly sensitive and rapid detection of formaldehyde vapor [69]. This chemiresistive sensor showed good sensitivity and can detect formaldehyde rapidly at ppb concentration levels. It is believed that the formation of weak and reversible charged intermediate complexes between the acid hydroxyl groups of TFQ and formaldehyde might account for the high sensitivity and selectivity of the sensor.

Xie et al. [70] fabricated a gas sensor with amino-functionalized MWCNTs on interdigitated electrodes. This sensor showed high selectivity in formaldehyde detection. The change of the relative resistance of the amino-modified sensor is up to 1.73% when the formaldehyde concentration is only 20 ppb. It also showed a quick response time of about 7–10 s, but its recovery time is relatively long. The interaction of MWNTs and amino-groups played a vital role for the excellent selectivity, low response time and great reproducibility of the gas sensor.

Wang et al. [71] functionalized SWCNTs by B-doping. When formaldehyde adsorption occurs 0.121 electrons were donated to the B-doped SWCNTs. Comparatively, only 0.021–0.039 electrons were donated to the pristine SWCNTs. The high sensitivity is ascribed to the strong chemical interactions between the formaldehyde (rich in electrons) and the B-doped SWCNT (lacking electrons).

Zhang et al. [72] investigated a SWCNT-Ag-LaFeO_3_ structure to detect formaldehyde. In this functionalized SWCNT film, formaldehyde reacts with the oxygen absorbed on LaFeO_3_ to form CO_2_ and H_2_O with the release of electrons, resulting in the thickening of the space-charge layer, thus increasing the potential barrier and decreasing the current. The LOD of this sensor is as low as 0.2 ppm for formaldehyde. The response time is 6 s and the recovery time is 20 s. Owing to these excellent properties, the SWCNT-Ag-LaFeO_3_ is a promising choice for the detection of formaldehyde.

Sensor arrays are also employed in the detection of formaldehyde. Lu et al. [73] fabricated a chemiresistive sensor array which included 32 sensor elements. Metal-loaded, polyethyleneimine functionalized and pristine SWCNTs are used as the sensing materials. An obvious response to formaldehyde was obtained when the concentration is as low as 10 ppb. 

Swager’s team has demonstrated a robust SWCNT-based chemiresistive sensor to selectively detect cyclohexanone, a target analyte for explosive, by functionalizing SWCNT with trifunctional selectors. The trifunctional selector noncovalently functionalized SWCNTs via cofacial π-π interactions, and it can binds cyclohexanone via hydrogen bond, and improves the overall robustness of SWCNT-based chemiresistors. Their sensors produced reversible and reproducible responses in less than 30 s to 10 ppm of cyclohexanone and displayed an average theoretical limit of detection (LOD) of 5 ppm [74].

## 10. CNT-Basedchemiresistive Sensors for Military and Defense Application

With the increased threat of terrorist activities, there is a need for the detection of explosives and chemical warfare agents, such as nitrotoluene, 2,4,6-trinitrotoluene (TNT) and the sarin simulant dimethyl methylphosphonate (DMMP). Li et al. [75] fabricated SWCNTs sensors for NO_2_ and nitrotoluene detection at room temperature. The electrical response is linear and reproducible. The detection limit was <44 ppb and 262 ppb for NO_2_ and nitrotoluene, respectively. The SWCNT networks or mesh were placed on an interdigitated electrode (IDE). The IDE was made by thermally evaporating 20 nm Ti and 40 nm Au on a SiO_2_ substrate. The SWCNT sensor showed a faster and reversible response toward nitrotoluene than NO_2_. Due to the higher bonding energy between SWCNTs and NO_2_, the recovery time was quite long (about ten hours). The response was weaker in sensing nitrotoluene because of the weak bonds and less electron transfer between SWCNTs and nitrotoluene molecules.

We functionalized SWCNT networks with 1-pyrenemethylamine (PMA). A chemiresistive sensor with high sensitivity was fabricated with the PMA-SWCNT networks for TNT detection. Negative charged complexes which can work as molecular gates were formed because of the selective interaction between TNT and amino substituent of PMA. The LOD for TNT was 10 ppt and the response time was less than 1 min [76]. Zhang et al. [77] prepared SWCNT chemiresistive sensors to monitor nitroaromatic explosives. The sensors were fabricated by the porous thin SWCNT films which coated with carbazolylethynylene (Tg-Car) oligomer. High sensitivity with a LOD at ppb levels was obtained.

We developed a high performance SWNT-TFQ (TFQ = Tetrafluorohydroquinone) network-based chamiresistive sensor to detect dimethyl methylphosphonate (DMMP) vapor [78]. The sensor is ultrasensitive, and can detect DMMP at the concentration of 20 ppt. The response time was less than 2 min. Besides the selectively interaction of DMMP with functionalized SWCNTs, the heavy hole doping of SWNTs by TFQ also contributes to the high sensitivity of the sensors. Table 3 summarizes the sensing performance of selected VOCs CNT-based chemiresistive sensors.

## 11. CNT-Based Biological Chemiresistive Sensors

Many types of biological sensors have been developed nowadays and CNT is one of the most popular sensing materials for the biosensors [79,80,81,82]. Cella et al. [83] reported a highly sensitive SWCNT chemiresistive sensor for the detection of some un-/weak-charged or small molecules. This sensor detected glucose via the displacement of plant lectin (concavalin A) which was bound to polysaccharide (dextran) immobilized on SWCNTs. This sensor exhibited picomolar sensitivity and selectivity toward glucose over human serum proteins and other sugars. A linear response correlation was obtained from 1 pM to 1 nM and had a sensitivity of 0.039 per pM glucose (the slope of the calibration plot). This biosensor is novel in that: (1) enzyme-free glucose detection with high selectivity and picomolar sensitivity is possible, (2) the adaptation of the displacement mode of biosensing to a chemiresistive sensor for detection of small molecules that would otherwise be difficult to detect by the chemiresistive/FET transduction principle. 

A high performance label-free SWCNT chemiresistive biosensor for the detection of human cardiac biomarker, myoglobin (Ag-cMb) was fabricated by Puri et al. [84] The electrophoretically aligned SWCNTs were electrochemically modified by poly(pyrrole-co-pyrrolepropylic acid) (PPy-PPa) with pendant carboxyl groups to immobilize highly specific cardiac myoglobin antibody (Ab-Mb). A concentration-dependent change in the source-drain current of the sensor towards the target, Ag-cM was observed in the range of 1–1000 ng mL^−1^ with a sensitivity of 118% per decadeb. This high sensitivity was attributed to high Ab-Mb probe loading of PPy-PPa copolymer, resulting in increased electron transfer to the SWNT on immunoreaction.

Sharma et al. [85] modified SWCNTs with platinum nanoparticles (PtNPs) and used this PtNP-SWNT chemiresistive sensor for label-free detection of myoglobin in phosphate buffer saline (PBS). The protein antibody, Ab-Mb, was covalently immobilized on PtNP attached over SWCNTs. Compared to bare SWCNT sensors (maximum response = 60.37%, dissociation constant = 26.62 ng mL^−1^), this funcionalized sensor has an enhanced response (dissociation constant = 111.14%) and a smaller dissociation constant (Kd = 19.98 ng mL^−1^). The response was in a concentration range of 0.1–1000 ng mL^−1^. The sensing mechanism of the chemiresistive sensor was explained by the increased charge scattering due to the antibody-antigen complex formation on immunoreaction, resulting in the reduction of charge carrier in p-type SWNT channel.

A chemiresistive sensor based on PtNP/SWNT hybrid was reported to detect human cardiac biomarkers troponin I (c TnI) in normal human serum [86]. The human cardiac biomarkers troponin I antibody (Ab-cTnl) was highly specific and it was fixed on the PtNP covalently via carboxyl groups. The source-drain current-voltage was measured to characterize the sensor and the changes of resistance toward different concentration of c TnI were recorded. A linear response was obtained in a concentration range from 0.001 ng mL^−1^ to 10 ng mL^−1^. A change of resistance per decade c TnI concentration is 15%. The transfer of electron mechanism was ascribed to the high sensitivity of the fuctionalized sensors.

Prakash et al. [87] reported an ultrasensitive chemiresistive biosensor for label-free detection of myoglobin. In this sensor, MWCNTs were embedded in the SU-8 nanofibers beyond the percolation limit in order to maximize the conductivity of nanofibers. In order to detect myoglobin, the aligned nanofiber was functionalized with the antibodies of myoglobin. A copper microelectrode array was fabricated on a glass substrate. The limit of detection is 6 fg/mL and a linear response was obtained from 20 fg/mL to 70 fg/mL. It is believed that binding of myoglobin on the funtionalized CNTs leads to increased surface stress on the nanofibers, which in turn increases the mobility of charge carriers in the MWCNTs, thus increasing the conductivity. Table 4 summarizes the sensing performance of selected CNT-based chemiresistive biosensors. 

## 12. Conclusion and Outlook

CNTs are an ideal nanomaterial for the development of solid-state sensing technology. The large aspect ratio and specific surface area make CNTs a highly sensitive sensing material and an efficient conducting channel. CNT-based sensors have been able to adapt to fields including industry, environmental protection, safety, medical testing, defense and the military. CNT-based chemiresisitive sensors can sense a variety of analytes, especially gases, and they can become a technology platform for the development of gas sensors. The most important current difficulties are how to further improve the sensitivity, selectivity and anti-jamming capability of CNT sensors. In order to improve the sensitivity and selectivity, present chemiresisitive sensors employ separation devices to capture and pre-concentrate analytes before sorption onto the sensor [86,87]. In the case of CNT-based chemiresisitive sensors, ultrahigh sensitivity is possible because analytes binding anywhere alone the nanotubes could disturb the whole conductance of the CNTs. This ultrahigh sensitivity makes use of the additional separation device to capture unnecessary analytes, which benefits miniaturization of the sensor system.

Researchers have been making unremitting efforts to improve the performance parameters of sensors. The current study focuses on reducing the LOD and improving the selectivity of the sensor to a practical analytical level. Stability is one of the important parameters when evaluating a sensor. The drift of the sensor is also an important parameter which can be partially solved by equipment calibration and data processing. Some metals, metal oxides and polymers can selectively interact with analytes, so if these substances are taken as the identification layer, selectivity may be enhanced. Also, the anti-jamming can be solved by wrapping CNTs with some functional materials that do not interact with the interference source. 

Although it is hard to anticipate the future impact of CNT-based sensors on the current sensor market, the most probable applications of CNT-based sensor technology are in the development of sensor arrays and compact, low-power, portable sensors. CNTs are easily integrated into microelectronic devices, so sensors with multiple micro-sensing arrays can be made. These multiple arrays allow sensors to analyze multiple analytes simultaneously while maintaining a small bulk. With the development of microelectronics technology, the cost of device manufacturing will decline, thus the commercialization of disposable sensors in a variety of application fields is distinctly possible. Additionally, traditional sensors work at high temperature, whereas CNT sensors can work at room temperature. This low energy consumption will lead to longer sensor battery lifetimes. Other advantages of CNT sensors include reduced sensor volume, long working life of the core component and sensor technology compatible with other microelectronics technology which makes possible the large scale preparation of sensor chips.

New trends in sensor technology are beginning to emerge. For example, new methods are required to collect more information from a single sensor. This goal might be achieved via development of multifunctional CNT sensor arrays combined with statistical methods. With the rapid development of communication and information technology and the internet of things, there is a need to combine wireless technology with sensors to produce large wireless sensor networks for accurate chemical mapping of large areas. CNT chemiresisitive sensors with high sensitivity, low power consumption, and easy miniaturization for massive sensor arrays could facilitate highly precise measurements and might find a place in such an opportunity.

## Figures and Tables

**Table 1:** Summary of sensing performance of chemiresisitive sensors.

Target Analytes	CNT Material/Method	LOD	Response Time	Rreversibility	Reference
NO_2_	Pristine	10 ppb	a few minutes	reversible	[43]
	polythiophene-SWNTs	10 ppb	low ~20 s	N/S	[45]
NH_3_	pristine	N/S	~180 s	reversible	[46]
	Au nanoparticles-decorated SWCNT	255 ppb	~20 s	N/S	[51]
	PANI-coated MWNT	0.2 ppm	10 s–120 s	N/S	[49]
	SWNT-PABS	5 ppm	~1 min	reversible	[53]
	PANI-SWNT network	50 ppb	sub-ppm	reversible	[54]

N/S = Not-stated.

**Table 2:** Summary of sensing performance of H_2_/CO_2_/CH_4_ chemiresisitive sensors.

Target Analytes	CNT Material/Method	LOD	Reference
H_2_	Pt-Pd-MWCNT	400 ppm	[57]
	Pd-MWCNT	2000 ppm	[57]
CO_2_	poly(ionic liquid) (PIL)-wrapped SWNTs	500 ppt	[61]
CH_4_	MWCNTs/ZnO composite	10 ppm	[62]

N/S = Not-stated.

**Table 3:** Summary of sensing performance of VOCs chemiresisitive sensors.

Target Analytes	CNT Material/Method	Detection Limit	Response Time	Reversibility	Reference
nitrotoluene	pristine	262 ppb	n/s	reversible	[75]
	SWNTs-poly(tetraphenylporphyrin) hybird	9 ppm	~8 min	reversible	[66]
TNT	PMA-SWCNT network	10 ppt	<1 min	n/s	[76]
formaldhyde	TFQ-functionalized SWNT	ppb level	< 1 min	n/s	[69]
	amino-functionalized MWCNTs	20 ppb	7–10 s	reversible	[70]
	SWCNT-Ag-LaFeO_3_ structure	0.2 ppm	6 s	reversible	[72]
cyclohexanone	trifunctional selectors functionalized SWCNTs	5 ppm	< 30 s	reversible	[74]
DMMP	SWNT-TFQ network	20 ppt	< 2 min	n/s	[78]

N/S = Not-stated.

**Table 4:** Summary of sensing performance of chemiresistive biosensors.

Target Analytes	CNT Material/Method	LOD/Concentration Range	Reference
glucose	polysaccharide immobilized SWNTs	50 nM for ConA cyclodextrin, 3.7 mM for dextran solution	[83]
myoglobin	poly(pyrrole-co-pyrrolepropylic acid) deposited SWCNT	1.0 ng mL^−1^ to 1000 ng mL^−1^	[84]
	PtNP-SWNT	0.1–1000 ng mL^−1^	[85]
	MWCTs embedded electrospun SU-8 nanofibers	LOD:6 fg/mL Range: 20 fg/mL to 70 fg/mL	[87]
c TnI	PtNP-SWNT	0.001 ng mL^−1^ to 10 ng mL^−1^	[86]

N/S = Not-stated.
